# Cellular pharmacodynamic effects of Pycnogenol® in patients with severe osteoarthritis: a randomized controlled pilot study

**DOI:** 10.1186/s12906-017-2044-1

**Published:** 2017-12-16

**Authors:** Steffen Jessberger, Petra Högger, Franca Genest, Donald M. Salter, Lothar Seefried

**Affiliations:** 1 Institut für Pharmazie und Lebensmittelchemie, Universität Würzburg, Am Hubland C7, 97074 Würzburg, Germany; 2 Department of Orthopedics, Universität Würzburg, Orthopedic Center for Musculoskeletal Research, Brettreichstraße 11, 97074 Würzburg, Germany; 3 Centre for Genomic and Experimental Medicine, University of Edinburgh, Osteoarticular Research Group, Crewe Road, Edinburgh, EH4 2XU UK

**Keywords:** Pycnogenol, Maritime pine bark extract, Osteoarthritis, Clinical study, ADAMTS, Cartilage, Synovial fluid, Serum, qPCR

## Abstract

**Background:**

The standardized maritime pine bark extract (Pycnogenol®) has previously shown symptom alleviating effects in patients suffering from moderate forms of knee osteoarthritis (OA). The cellular mechanisms for this positive impact are so far unknown. The purpose of the present randomized pilot controlled study was to span the knowledge gap between the reported clinical effects of Pycnogenol® and its in vivo mechanism of action in OA patients.

**Methods:**

Thirty three patients with severe OA scheduled for a knee arthroplasty either received 100 mg of Pycnogenol® twice daily or no treatment (control group) three weeks before surgery. Cartilage, synovial fluid and serum samples were collected during surgical intervention. Relative gene expression of cartilage homeostasis markers were analyzed in the patients’ chondrocytes. Inflammatory and cartilage metabolism mediators were investigated in serum and synovial fluid samples.

**Results:**

The oral intake of Pycnogenol® downregulated the gene expression of various cartilage degradation markers in the patients’ chondrocytes, the decrease of MMP3, MMP13 and the pro-inflammatory cytokine IL1B were statistically significant (*p* ≤ 0.05). Additionally, protein concentrations of ADAMTS-5 in serum were reduced significantly (p ≤ 0.05) after three weeks intake of the pine bark extract.

**Conclusions:**

This is the first report about positive cellular effects of a dietary supplement on key catabolic and inflammatory markers in patients with severe OA. The results provide a rational basis for understanding previously reported clinical effects of Pycnogenol® on symptom scores of patients suffering from OA.

**Trial registration:**

ISRCTN10754119. Retrospectively registered 08/10/2015.

## Background

Osteoarthritis (OA) is a highly prevalent degenerative joint disease causing pain, joint stiffness and disability [1]. It has been estimated that by the year 2030 more than 20% of the adults will suffer from OA in the western countries [2] which leads to increasing global health costs. Current pharmacologic treatment only allows management of symptoms without effects on disease progression and may be associated with potential adverse side effects [3]. The perceived burden of suffering often prompts patients to seek therapeutic alternatives, e.g. plant-derived remedies. Dietary factors or supplements have been discussed as options in the management or prevention of OA [4] and their potential as chondroprotectives has been investigated [5].

A dietary supplement which has shown clinical effects in patients with mild forms of knee OA (Kellgren-Lawrence grade I and II) is the standardized maritime pine bark extract Pycnogenol® [6–8]. In three double-blind randomized placebo-controlled studies with 37–159 patients receiving 100 mg [7] or 150 mg [6, 8] Pycnogenol® per day over three months, statistically significant reductions of the composite Western Ontario and McMaster Universities (WOMAC) score summarizing pain, joint stiffness and daily activities were observed. Additionally, a decrease in the concomitant use of analgesic drugs was reported. The cellular mechanisms responsible for the positive effects of Pycnogenol® on the clinical symptoms are unknown.

The pine bark extract is a complex mixture of polyphenolic compounds [9]. A pharmacokinetic study with volunteers ingesting Pycnogenol® revealed that catechin, caffeic acid, ferulic acid, and taxifolin were detectable in a nanomolar range in plasma [10]. Moreover, a catechin metabolite (M1) produced by human intestinal bacteria was found in the plasma samples. Subsequent investigations showed that M1 exerted various anti-inflammatory effects in vitro such as the inhibition of the activity of the matrix metalloproteinases MMP-1, −2 and −9, decrease of the release of MMP-9 from human monocytes or inhibition of the expression of the inducible NO synthase (iNOS) in RAW 264.7 macrophages [11, 12]. In other in vitro experiments using the whole extract a decrease of IL1B mRNA synthesis in RAW 264.7 cells was reported [13] as well as inhibitory effects on the expression of COX-2, IL-8 and iNOS in human chondrocytes and fibroblasts [14]. However, since not all components of the extract are bioavailable and other bioactive molecules such as M1 are generated in vivo, it is not clear whether experiments using the whole extract would be indicative for cellular effects that actually occur in vivo.

The results of in vitro and ex vivo approaches with the maritime pine bark extract are interesting in the context of the pathology of OA which is characterized by loss of articular cartilage and remodeling processes, pain and functional impairment [15, 16]. Destructive effects are related to an increase of cartilage catabolism while anabolic processes are suppressed. Various biomarkers are upregulated in OA patients and can be detected in serum or plasma samples, synovial fluid or urine. The pro-inflammatory cytokine IL-1β actives various signal transduction pathways e.g. via NF-κB that result in upregulation of pro-inflammatory mediators and cartilage degrading proteolytic enzymes such as MMP-1, MMP-3 and MMP-13 [15]. MMPs degrade collagen and aggrecan, the major proteoglycan in human articular cartilage. Aggrecan is also targeted by another class of metalloproteinases, the aggrecanases-1 and -2, also known as “A Disintegrin and Metalloproteinase with Thrombospondin Motifs” (ADAMTS4, −5) [16, 17]. The role of different ADAMTS is not definitely resolved so far. In mice, ADAMTS-5 seems to be the most important enzyme degrading aggrecan molecule [18]. ADAMTS5 knockout mice are protected from cartilage loss, whereas ADAMTS4-KO mice are not [19]. In humans, both enzymes play a role in the pathophysiology of OA [17, 20]. Cartilage degradation by MMPs, ADAMTS and also cathepsins generates various decomposition products such as type II collagen fragments, CTX-II (carboxy-terminal telepeptides of type II collagen) and Helix-II (type II collagen helical peptide) [21]. Aggrecan depletion is indicated by sulfated glycosaminglycans (sGAG) [17].

So far research insights regarding the cellular effects of plant-based extracts or single constituents on relevant OA biomarkers have been gained from in vitro cell culture studies and experiments with animals receiving peroral or intra-articular treatment [5, 22]. To the best of our knowledge there has been no approach reported which involves OA patients taking a plant extract and determines effects on biomarkers simultaneously in the participants’ articular cartilage, synovial fluid and serum. The purpose of the present pilot study was to span the knowledge gap between the reported clinical efficacy of Pycnogenol® and its in vivo mechanism of action in OA patients.

## Methods

### Patients and study design

The present study was a randomized controlled clinical trial involving patients suffering from severe osteoarthritis (OA) of the knee according to the WOMAC score who were scheduled for an elective arthroplasty (Kellgren-Lawrence grade III-IV). Patients were not eligible if they regularly took NSAIDs or glucocorticoids p.o. within the past four weeks, if they currently received a therapy with anti-coagulants or if they were tested positive for HIV, HCV or HCB or if they had a previous or current infection of the affected knee joint. As rescue medication acetaminophen (paracetamol), tramadol or a combination of tilidine and naloxone was allowed. The study protocol was reviewed and approved (reference number 248/11) by the local Ethics Committee of the Medical Faculty of the University Würzburg. The procedures followed were in accordance with the ethical standards of the Ethics Committee and with the Helsinki Declaration of 1975, as revised in 2000. Since the study primarily focused on pharmacokinetic/bioanalytical aspects (see [23]) an early registration was overlooked and the study was registered retroactively. The authors confirm that all their ongoing and related trials performed for this drug/intervention are registered.

A total of 33 OA patients were recruited for the study and gave written informed consent. The number of participants was chosen based on an earlier pharmacokinetic study with healthy volunteers [10]. With respect to cellular pharmacodynamic effects the present study was of exploratory nature due to lack of reference data. The insights generated can be used as rational basis for future sample size calculations in similar study settings. Patients were randomized into two groups using a computer-generated randomization list which was not accessible to the physicians and nurses who were involved in the patient care and management. Study participants were either assigned to the treatment group (*n* = 16) receiving 200 mg Pycnogenol® (Horphag Research Ltd., Geneva, Switzerland) per day (twice daily two capsules with each 50 mg) over three weeks prior to the planned surgery or to the control group (*n* = 17) who received no Pycnogenol®. All patients were asked to refrain from polyphenol-rich food/beverages. Nutritional check-lists were provided for specifying food/beverages to avoid and for recording ingested items within the last two days before blood sampling. Adherence to the study medication was estimated based on counting the number of returned Pycnogenol® capsules. Patients who ingested more than 80% of study medication were considered to be adherent.

Blood samples were collected (BD Vacutainer® SST II Advance; 13 mm × 100 mm; Becton Dickinson GmbH, Heidelberg, Germany) before oral intake of Pycnogenol® (V1, basal value); during the intake, approximately 1–2 days before the surgery (V2); and during or shortly before knee surgery (V3), about 12 h after the last dose of Pycnogenol®. On the day of the surgery residual knee cartilage and synovial fluid were also collected. The knee fragments were stored in DMEM for 0.5 to 1 h until further processing. Serum and synovial fluid samples were shock-frozen and stored at −80 °C. The outcome measures were the expression / concentrations of various inflammatory and degradations markers in chondrocytes, synovial fluid and serum.

All medical procedures including enrollment of participants, surgery, patient care and sample collection took place at the orthopedic center (Orthopädie und Orthopädische Klinik König-Ludwig-Haus, Universität Würzburg) between September 2012 and September 2014. The generation of the random allocation sequence, assignment of participants to the intervention or control group and analysis of all patient samples took place at the Institut für Pharmazie und Lebensmittelchemie.

### Chemicals and reagents

Buffers and cell culture media were all obtained from Sigma Aldrich (Taufkirchen, Germany). Fungizone®, Type-II-Collagenase and recombinant human interleukin-1β were purchased from Life Technologies (Darmstadt, Germany). Fetal bovine serum (FBS), Trypan blue solution 0.5% [m/v] and L-glutamine (200 mM) were obtained from Biochrom AG (Berlin, Germany). All disposable items used in cell culture or sample handling were purchased from Sarstedt AG (Nümbrecht, Germany) or Greiner-bio one (Frickenhausen, Germany).

### Treatment of cartilage

The complete residual articular cartilage was removed from the patients’ samples to analyze the average expression of marker genes in the chondrocytes. The cartilage was cut into 1–2 mm^2^ fragments and placed in an antimicrobial solution containing 5 mL Fungizone® and 5 mL L-glutamine (200 mM) in 100 mL Dulbecco’s phosphate buffered saline (PBS) for 30 min at room temperature. The digestion procedure has been described elsewhere [14]. Briefly, the pieces were washed three times with PBS and digested with trypsin 0.25% / EDTA 0.02% for 30 min at 37 °C and 5% CO_2_. After repeating the washing step the cartilage fragments were incubated with a solution of 0.3% (m/v) type-II collagenase in IMDM over night at 37 °C and 5% CO_2_. Then the cell suspension was filtered through an autoclaved metal wire (VWR, Darmstadt) to remove remaining large cartilage fragments. After centrifuging the suspension at 1560 *g* for 10 min at room temperature the cell pellet was suspended and washed in 10 mL of PBS. This washing step was repeated twice. The cell pellet was resuspended in 7.5 mL IMDM and filtered through a 70 μm cell sieve. The cell yield was determined using a Neubauer counting chamber after 1:1-dilution of the suspension with trypan blue solution 0.5% (m/v). Cell aliquots of approximately 1 × 10^6^ cells were shock-frozen in liquid nitrogen and stored at −80 °C. Only cell samples showing >95% viability were used for further experiments.

### RNA extraction and cDNA synthesis

Total RNA from human primary chondrocytes was extracted using a high pure RNA isolation kit (Roche Diagnostics, Mannheim, Germany). Concentration and purity of the resulting RNA was analyzed with an Infinite® 200 PRO NanoQuant (TECAN Ltd. Group, Männedorf, Switzerland) using the i-control™ Microplate Plate Reader software, version 1.10 (TECAN Ltd. Group). Only samples with a ratio of A_260nm_/A_280nm_ between 1.9 and 2.1 were accepted. For first strand complementary DNA synthesis, 250 ng, 500 ng or 1 μg of total RNA, dependent on the yield, were transcribed into cDNA in a two-step process with a Transcriptor First Strand cDNA synthesis kit (Roche Diagnostics) according to the manufacturer’s instructions and using oligo dT-primers.

### Quantitative real-time polymerase chain reaction (qPCR)

**Fig. 1:**
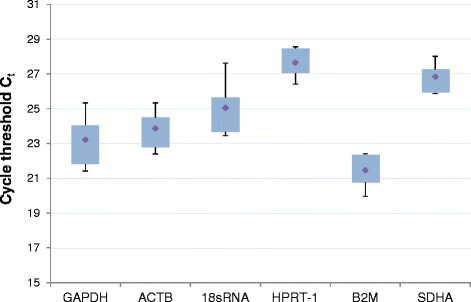
Housekeeping gene validation data from five cartilage samples obtained from patients suffering from severe knee osteoarthritis. The genes HPRT-1 and SDHA were identified as the most stable ones using the BestKeeper™-software. ACTB was preferred over B2M due to its frequent use in literature

Complementary DNAs were amplified under following conditions: 95 °C for 3 min, 40 cycles of 95 °C for 10 s and 60 °C for 30 s using the iTaq™Universal SYBR®Green-MasterMix (BioRad Laboratories GmbH, München, Germany) and a Stratagene Mx3005P qPCR system (Agilent Technologies, Waldbronn, Germany) with MxPro-Software, version 4.10 (Agilent Technologies). Specific primers were designed with the PrimerBLAST™ software for each target and housekeeping gene and were obtained from Eurofins MWG GmbH (Ebersberg, Germany) (Table 1). The qPCR mix contained 10 μL SYBR®Green Master Mix, 2 μL of a 1:10-dilution of cDNA and varying amounts of primers and water up to a final volume of 20 μL. Optimal primer concentrations were determined using a primer matrix (Table 2). During housekeeping gene validation the gene expression of β-actin (ACTB) and hypoxanthine phosphoribosyl transferase 1 (HPRT-1) were identified as the most stable reference genes under the chosen conditions (*n* = 5; Fig. 1). Mean efficiencies of amplification processes ranged from 92% to 99% (Table 2). Amplification efficiencies of all genes and C_t_-values of target and reference genes were included in the calculations using the REST® 2009-software [24]. A melting curve analysis was performed after each run to control the specificity of the amplification process.

**Table 1:** Specific primers used in qPCR

Gene	Type	Size [bp]	Sequence [5' → 3']
GAPDH	forward	20	CGC TCT CTG CTC CTC CTG TT
GAPDH	reverse	20	CCA TGG TGT CTG AGC GAT GT
ACTB	forward	18	TGA GCG CGG CTA CAG CTT
ACTB	reverse	22	TCC TTA ATG TCA CGC ACG ATT T
18sRNA	forward	20	GCC TGC GGC TTA ATT TGA CT
18sRNA	reverse	20	ACC AGA CAA ATC GCT CCA CC
HPRT-1	forward	22	AGC CAG ACT TTG TTG GAT TTG A
HPRT-1	reverse	21	ACT GGC GAT GTC AAT AGG ACT
B2M	forward	25	AAG ATA GTT AAG TGG GAT CGA GAC A
B2M	reverse	23	AAT TCA TCC AAT CCA AAT GCG GC
SDHA	forward	22	AGA CCT AAA GCA CCT GAA GAC G
SDHA	reverse	24	CTC ATC AAT CCG ACC TTG TAG TC
MMP1	forward	20	TGG ACC TGG AGG AAA TCT TG
MMP1	reverse	20	GGT ACA TCA AAG CCC CGA TA
MMP3	forward	24	AGG CAA GAC AGC AAG GCA TAG AGA
MMP3	reverse	24	ACG CAC AGC AAC AGT AGG ATT GGA
MMP13	forward	22	TGG AAT TAA GGA GCA TGG CGA C
MMP13	reverse	20	ACC TAA GGA GTG GCC GAA CT
IL1B	forward	24	AAT CTC CGA CCA CCA CTA CAG CAA
IL1B	reverse	24	AAG GGA AAG AAG GTG CTC AGG TCA
ADAMTS5	forward	24	ACA AGA GCC TGG AAG TGA GCA AGA
ADAMTS5	reverse	24	TGA TGC CCA CAT AAA TCC TCC CGA
CTSK	forward	20	TTC CCG CAG TAA TGA CAC CC
CTSK	reverse	20	GGA ACC ACA CTG ACC CTG AT

**Table 2:** Optimal primer concentrations and amplification efficiencies of each target gene

Gene	Concentration forward primer [μM]	Concentration reverse primer [μM]	Amplification efficiency [%]
GAPDH	0.15	0.15	–
ACTB	0.15	0.15	92.97
18sRNA	0.15	0.15	–
HPRT-1	0.15	0.15	96.93
SDHA	0.15	0.15	93.40
B2M	0.375	0.375	–
MMP1	0.75	0.75	96.77
MMP3	0.50	0.50	99.27
MMP13	0.75	0.75	95.27
IL1B	0.50	0.50	91.97
ADAMTS5	0.75	0.75	96.97
CTSK	0.25	0.25	99.87

### Determination of marker concentrations in serum and synovial fluid

Human MMP-1 and MMP-13 ELISA kits were purchased from RayBiotech® (Norcross, GA, USA). Human ADAMTS-4 ELISA kit was obtained from Novateinbio (Woburn, MA, USA) and human ADAMTS-5 ELISA kit was purchased from Cusabio (Wuhan, China). Human MMP-3 CytoSet™ and Antibody Pair Buffer Kit were produced by Invitrogen Corporation (Frederick, USA). The synovial fluid samples were centrifuged at 1000 *g* for 10 min using the Megafuge 1.0 R Thermo Scientific (Waltham, MA, USA). The assays were then performed according to the manufacturers’ protocols.

### Dimethyl-methylene blue (DMMB) assay

Shark chondroitin sulfate, used as a standard, DL-dithiothreitol (DTT), papain from *papaya latex*, iodacetic acid and DMMB were obtained from Sigma Aldrich. A calibration curve in a range of 0.25–5 μg chondroitin sulfate in water was obtained (UV mini-1240; Shimadzu, Duisburg, Germany) for calculating the concentrations of sulfated glucosamine glycans (sGAG) in synovial fluid samples of patients according to Farndale et al. [25]. Briefly, synovial fluid samples were diluted 1:10 with a solution of 1 mM Na_2_H_2_EDTA, 2 mM DL-DTT and 300 μg/mL papain in 20 mM sodium phosphate buffer (pH = 6.8) to a final volume of 1 mL. The mixture was incubated for 1 h at 60 °C and 300 rpm in a thermomix (Eppendorf, Hamburg, Germany). The reaction was stopped with 1 mL 20 mM iodacetic acid and diluted with 3 mL of 50 mM TRIS/HCl buffer (pH = 8.0). 500 μL of this incubation mix were added to 2 mL of a dimethylenblue solution and the absorption was measured at λ = 525 nm.

### Statistical methods

Relative gene expressions and statistical analysis of gene expression differences among the groups were calculated using the REST2009® software [24]. Housekeeping genes were evaluated with the BestKeeper® software [26]. This software uses the standard deviation as measure for gene expression stability with a cut-off of +/− 1 C_p_ value. All other mathematical operations were performed using the GraphPad® Prism software, version 6.0 (GraphPad Software, Inc., La Jolla, CA, USA). Intergroup differences were calculated with the Student’s t-test, correlations were analyzed using the Pearson’s correlation test. Statistical significance was defined as *p*-values ≤0.05.

## Results

### Patients and protocol adherence

**Fig. 2:**
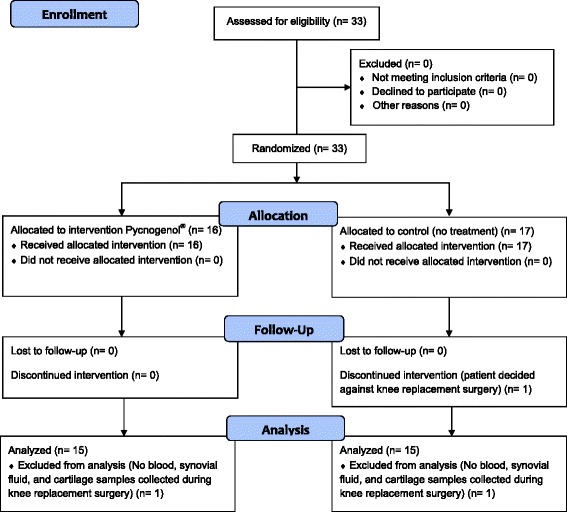
CONSORT 2010 Flow diagram of the study (see http://www.consort-statement.org/)

A total number of 33 patients were enrolled into the study and randomized to receive either Pycnogenol® (*n* = 16) or no treatment (*n* = 17). One patient of the control group decided against the scheduled knee replacement surgery and was excluded. During the surgical procedure blood, synovial fluid and cartilage samples were failed to collect for one patient each of the control and Pycnogenol® group. These patients were excluded from the analysis (Fig. 2). Thus, 30 patients were evaluated, 15 in the treatment group (9 females, 6 males) and 15 in the control group (11 females, 4 males; Table 3). There was no statistically significant difference between the groups in any of the basic demographic characteristics (Student’s T-test, *p* > 0.05). Analysis of the nutrition protocols revealed that the dietary advice was not followed well and violations were admitted before collecting 42% of the blood samples. In contrast, the adherence to the study medication was excellent based on pill count-back of the returned medication containers. All but one study participant (#130, Pycnogenol® group; 76%) fulfilled the adherence criteria. Without this patient, the average adherence was 99.4% (range 96–100%). No treatment-associated adverse effects were reported except for one patient of the Pycnogenol® group who experienced flatulence.

**Table 3:** Basic demographic characteristics of the patients participating in the study

Parameter	Pycnogenol® group	Control group	Total
Number of patients	15	15	30
Age [years]	63.3 ± 7.6	65.3 ± 8.8	64.3 ± 8.2
Body height [m]	1.69 ± 0.10	1.69 ± 0.11	1.69 ± 0.10
Body weight [kg]	90.22 ± 15.95	79.90 ± 24.12	87.33 ± 15.66
BMI [kg/m^2^]	30.87 ± 4.72	28.98 ± 4.14	30.74 ± 5.29
Percentage of women [%]	60	73.3	66.7

### Influence of Pycnogenol® on gene expression in patients’ chondrocytes

All cDNA samples investigated for relative expression of target genes were examined regarding the reliability of the housekeepers using the BestKeeper® software [26]. Hypoxanthine phosphoribosyltransferase 1 (HPRT-1) and beta actin (ACTB) were confirmed as reliable housekeeping genes with standard deviations of the expression below +/− 1 C_p_ value (Table 4; Fig. 1). Using two reference genes complies with the latest MIQE guideline for qPCR analysis [27] . Further calculations were done without patient #119 as a statistically confirmed outlier regarding MMP-3 expression and patient #130 who showed less than 80% adherence to the study medication (Table 4).

**Table 4:** Relative gene expressions of cartilage homeostasis markers in chondrocytes of patients treated with Pycnogenol® compared to controls

Target gene	Exclusion	Relative expression [95% CI]	*P*-value
MMP1	none	0.234 [0.005; 10.058]	0.15
MMP3	none	0.442 [0.087; 1.977]	0.07*
MMP3	#119	0.367 [0.026; 3.552]	0.02**
MMP3	#130	0.394 [0.025; 13.198]	0.04**
MMP3	#119, #130	0.315 [0.023; 3.001]	0.01**
MMP13	none	0.265 [0.018; 3.460]	0.06*
MMP13	#130	0.292 [0.003; 8.698]	0.05*
IL1B	none	0.376 [0.059; 2.423]	0.08*
IL1B	#130	0.359 [0.013; 7.112]	<0.05**
ADAMTS5	none	1.030 [0.305; 3.793]	0.94
CTSK	none	0.622 [0.143; 2.991]	0.25

Further calculations were done without patients #119 as a statistically confirmed outlier regarding MMP3 expression and #130 who showed less than 80% adherence to the study medication

*tendency to down-regulation

**statistically significant (*p* ≤ 0.05)

**Fig. 3:**
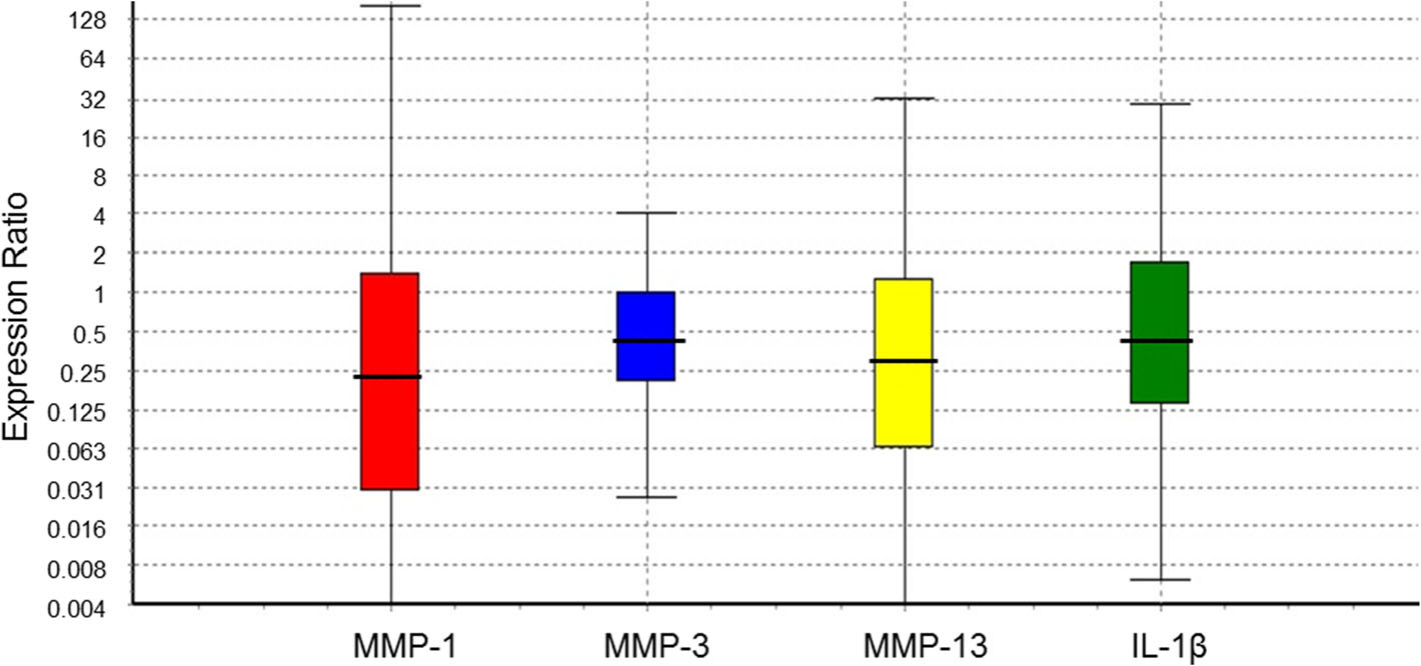
Relative gene expression of several marker genes of cartilage homeostasis. The relative gene expression of targets was calculated by dividing the expression of the gene of interest by the geometric mean of the expression of the reference genes. All target genes showed a tendency to downregulation in the Pycnogenol® group (*n* = 14) in relation to the control group (*n* = 14). The relative gene expression of the particular targets in the control group is referred to as “1”. Calculations and box-and-whisker-plots were made with the REST2009® software. The dashed lines in the boxes symbolize the median of the relative expressions

There was a clear tendency towards downregulation of the gene expression of the matrix metalloproteinases MMP1, MMP3 and MMP13 in the Pycnogenol® group, although the effect was not always statistically significant due to the high variability and the limited patient number (Table 4; Fig. 3). MMP1 was downregulated to a ratio of 0.234 [0.005; 10.058]. The relative gene expression of MMP3 was decreased to 0.442 [0.087; 1.977] and to 0.367 [0.026; 3.552] (*p* = 0.02) when excluding patient #119 (control group) as an outlier regarding MMP3 expression. The additional exclusion of the non-adherent patient #130 made the downregulation of MMP3 even clearer (0.315 [0.023; 3.001] (*p* = 0.01)). MMP13 was downregulated by Pycnogenol® to a ratio of 0.265 [0.018; 3.460]. Exclusion of the non-adherent patient #130 resulted in a statistically significant difference between the study groups (0.292 [0.003; 8.698], *p* = 0.05).

The relative gene expression of IL1B was downregulated to a ratio of 0.376 [0.059; 2.423]. Excluding patient #130 due to non-adherence the tendency to down-regulation was statistically significant (*p* < 0.05). Analysis of ADAMTS5 gene expression (1.030 [0.305; 3.793]) did not reveal any change after intake of Pycnogenol®.

### Inflammatory and cartilage metabolism markers in synovial fluid

The synovial fluid sample volumes of three patients of the Pycnogenol® group (#108, #120 and #121) and one of the control group (#106) were not sufficient to determine all biomarkers of interest. Additionally, depending on the target, the concentrations in some samples were below the detection limit of the ELISA kits so that the results frequently included less than 15 patients per study group (Table 5).

**Table 5:** Concentrations of various inflammatory and cartilage metabolism protein markers in the synovial fluid of patients treated with Pycnogenol® compared to controls

Target	Pycnogenol® group (n)	Control group (n)	*P*-value
MMP-1	11.61 ± 8.61 ng/mL (9)	14.04 ± 6.93 ng/mL (12)	0.47
MMP-3	7.06 ± 2.46 ng/mL (12)	7.10 ± 2.72 ng/mL (13)	0.96
MMP-13	0.23 ± 0.13 ng/mL (13)	0.27 ± 0.18 ng/mL (14)	0.51
CTX-II	236.76 ± 168.08 pg/mL (12)	428.90 ± 511.83 pg/mL (14)	0.23
Helix-II	0.90 ± 0.68 nmol/L (12)	1.23 ± 1.19 nmol/L (15)	0.57
sGAG	356.89 ± 61.89 μg/mL (12)	390.32 ± 53.81 μg/mL (15)	0.19
ADAMTS-4	32.50 ± 33.26 ng/mL (9)	22.55 ± 19.69 ng/mL (12)	0.42
ADAMTS-5	12.58 ± 10.83 ng/mL (12)	7.08 ± 4.28 ng/mL (15)	0.12

Concentrations of matrix-degrading enzymes of the MMP family were not clearly reduced in the Pycnogenol® group while both ADAMTS-4 and ADAMTS-5 proteins were found at higher concentration in the intervention group compared to controls (Table 5). However, none of the differences was statistically significant (*p* > 0.05).

The release of sulfated glycosaminoglycans (sGAG) into synovial fluid was lower in the group treated with Pycnogenol® compared to untreated controls although this effect did not reach statistical significance (Table 5). Type II collagen fragments, CTX-II (carboxy-terminal telepeptides of type II collagen) and Helix-II (type II collagen helical peptide), were reduced in the Pycnogenol® group, but the high inter-individual variability again impeded statistical differentiation of the groups.

### Alteration of cartilage metabolism markers in serum

Serum samples from each patient were obtained both before (V1) and after (V2 and V3) intake of Pycnogenol®. V3 samples were primarily used for analysis of Pycnogenol® constituents and metabolites [23].

**Fig. 4:**
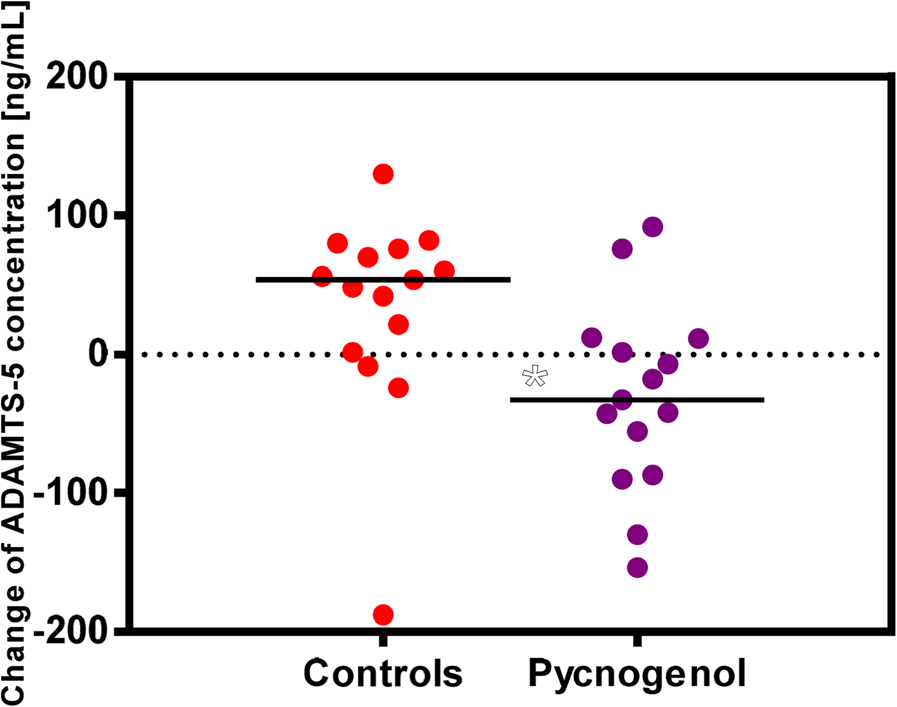
Changes of ADAMTS-5 protein concentrations in serum of the study participants from V1 to V2 in the Pycnogenol® group (*n* = 15) in relation to controls (*n* = 15). For each patient the ADAMTS-5 concentration after three weeks of intervention (V2) was subtracted by the concentration before the intervention (V1). Each dot represents a single patient. The difference between the two groups was statistically significant (*p* = 0.02), which was even more pronounced after exclusion of one outlier in the control group (*p* = 0.001)

There was a significant difference in the changes of protein concentrations of ADAMTS-5 in serum from V1 to V2 between the Pycnogenol® treated group (*n* = 15) compared to the control group (n = 15) (*p* = 0.02; Fig. 4). The mean decline of 31.12 ± 67.85 ng/mL ADAMTS-5 in the Pycnogenol® group was opposed by an increase of 33.37 ± 72.82 ng/mL in the control group. After excluding a statistically confirmed outlier in the control group (patient #132; Δc = −188 ng/mL; identified by GraphPad® Prism Software), the difference among the groups was even more pronounced (*p* = 0.001).

**Fig. 5:**
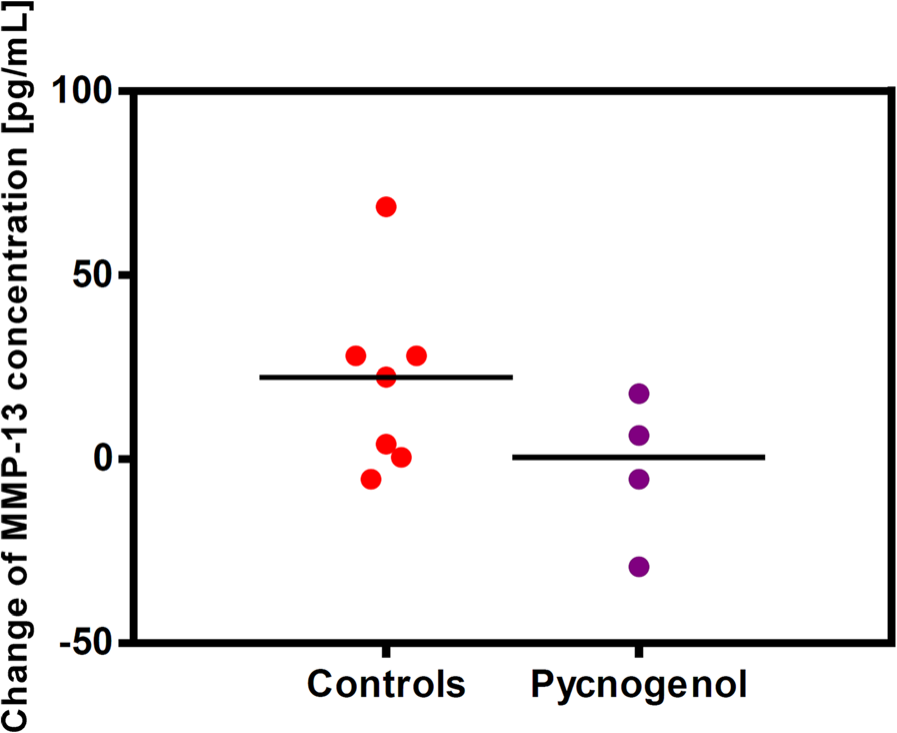
Changes of MMP-13 protein concentrations in the patients’ serum samples from V1 to V2 in the Pycnogenol® group (*n* = 4) compared to controls (*n* = 7). For each patient the MMP-13 concentration after three weeks of intervention (V2) was subtracted by the concentration before the intervention (V1). Each dot of the scatter plot represents a single patient. The difference between the groups was not statistically significant which was also due to not quantifiable levels of MMP-13 in serum samples of 19 patients (*p* = 0.15)

The analysis of MMP-13 concentrations revealed a similar trend (Fig. 5). There was a minor decrease of 2.62 ± 20.17 pg/mL MMP-13 in the Pycnogenol® group (*n* = 4) compared to a slight increase of 20.87 ± 25.09 pg/mL in control patients (*n* = 7). The difference between the groups was not statistically significant (*p* = 0.15). The MMP-13 serum concentrations of 19 patients were below the lower limit of detection of the ELISA and therefore excluded from calculations.

There were no relevant changes of the serum concentrations of other markers between V1 and V2 in any of the two groups (data not shown).

### Correlation analysis of biomarkers

**Fig. 6:**
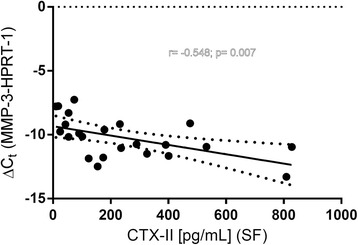
Correlation between the MMP3 gene expression and the concentration of CTX-II in synovial fluid (SF) of patients. Gene expression is expressed as ΔC_t_ (C_t_ (MMP3)-C_t_ (HPRT-1)). Higher expression levels of MMP3 in cartilage resulted in higher CTX-II levels in synovial fluid. The correlation was statistically significant (*r* =-0.548, *p* = 0.01; *n* = 23). The dotted lines symbolize the 95% CI

The ΔC_t_ values of the MMP3 expression in relation to the housekeeping gene HPRT-1 statistically significantly correlated with the concentration of CTX-II in synovial fluid (SF) (*r* = −0.548, *p* = 0.01; *n* = 23). Thus, higher gene expression levels of MMP3 in cartilage resulted in higher CTX-II levels in synovial fluid (Fig. 6). A similar correlation was seen for the expression of MMP1 and CTX-II. The correlations were statistically significant using ACTB or HPRT-1 as reference genes (*p* = 0.049 and *p* = 0.042, *n* = 23).

**Fig. 7:**
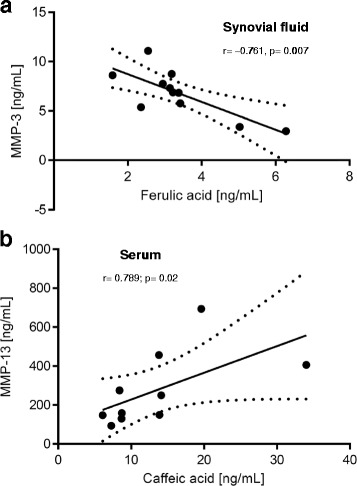
Correlations between concentrations of polyphenolic constituents of Pycnogenol® and matrix metalloproteinases (MMPs). **a.** Inverse correlation between concentrations of MMP-3 protein and ferulic acid in the patients’ synovial fluid. This correlation was statistically significant (*r* = 0.761, *p* = 0.007; *n* = 11). **b.** Direct relationship between MMP-13 protein concentrations and levels of caffeic acid in serum (r = 0.789, *p* = 0.02; n = 8). The dotted lines symbolize the 95% CI

**Fig. 8:**
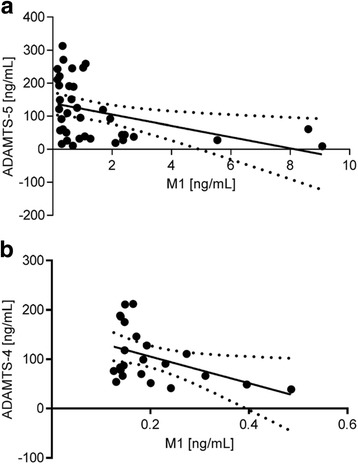
Correlations between serum / blood cell concentrations of a microbial metabolite (M1) of Pycnogenol® and ADAMTS protein concentrations in serum of the patients. **a**. Relationship between concentration of M1 and ADAMTS-5 levels in serum (*n* = 23). The inverse correlation was weak, but statistically significant (r = −0.395; *p* = 0.016). **b**. Relationship between concentration of M1 in blood cells and ADAMTS-4 levels in serum (n = 23). The inverse correlation was weak, but statistically significant (r = −0.445; *p* = 0.033). The dotted lines symbolize the 95% CI

Effect parameters were also correlated with concentrations of the constituents of Pycnogenol® and its metabolite M1 in serum, synovial fluid and in blood cells [23]. Higher concentrations of ferulic acid in synovial fluid significantly correlated with lower MMP-3 protein levels in this specimen (*r* = −0.761, *p* = 0.007; *n* = 11; Fig. 7a). In contrast, a significant direct correlation was found between caffeic acid and MMP-13 concentrations in serum of the study participants (*r* = 0.789, *p* = 0.02; *n* = 8; Fig. 7b). Weak, but statistically significant inverse correlations were found for concentrations of M1 and ADAMTS-5 in serum of the OA patients (*r* = −0.395, *p* = 0.016; *n* = 23; Fig. 8a) and between M1 concentrations in blood cells and ADAMTS-4 levels in serum (*r* = −0.445, *p* = 0.033; *n* = 23; Fig. 8b).

## Discussion

This is the first report of a randomized controlled clinical study on the cellular effects of the maritime pine bark extract Pycnogenol® on various catabolic and inflammatory markers in patients with severe osteoarthritis (OA) undergoing a medically indicated knee replacement surgery. Patients with end-stage OA were chosen since this was an ethical option to simultaneously obtain cartilage samples and synovial fluid along with blood samples. The recruited participants were considered as representative OA patients with a mean age of 64.3 ± 8.2 years and an elevated BMI of 30.74 ± 5.29 kg/m^2^ [28]. Above the age of 60 years approximately twice as many women (18%) suffer from OA compared to men (9.6%) [29]. This relation was also mirrored in the present study population which included 20 women and 10 men. After randomization half of the recruited patients received Pycnogenol® over three weeks prior to joint replacement surgery. In clinical studies previously investigating the effects Pycnogenol® on clinical symptoms the patients were treated over three months [6–8]. All studies revealed time-dependent effects on symptoms of OA, earliest statistically significant differences between Pycnogenol® and placebo regarding the overall WOMAC score were observed after six weeks [8]. Since an improvement of OA symptoms is typically a “late” event we hypothesized that changes on a cellular level would occur earlier. The three weeks of Pycnogenol® intake in the present study were chosen on the basis of the time period that the clinicians routinely scheduled between pre-admission meetings with the patients and the surgery. During this meeting a basic examination was performed, the patients were supplied with the pine bark extract and pre-intervention blood samples were collected.

Despite of the fact that three weeks of Pycnogenol® intake were comparatively short the expression analysis in the articular chondrocytes revealed for all but one parameter a downregulation of the respective gene in the Pycnogenol® group compared to controls. The decreased expressions of MMP3, MMP13 and IL1B genes were statistically significant when single factors contributing excessively to the data variability were excluded based on statistical procedures or documented non-adherence. A concomitant downregulation of IL1B and the MMPs is consistent with the accepted belief that the IL-1β protein is a strong inducer of MMP1, MMP3 and MMP13 expression as well as inducing its own upregulation [15]. In contrast, the aggrecanase ADAMTS5 was not downregulated suggesting that its expression in the patients’ articular chondrocytes was not or not entirely dependent on IL-1β exposure. Indeed, there are contradicting reports about the induction of ADAMTS5 by IL-1β [15, 17]. The proteolytic cysteine protease cathepsin K (CTSK) which contributes to collagen and aggrecan degradation [30] was downregulated in the Pycnogenol® group, but this effect did not reach statistical significance. Whilst numerous in vitro or in vivo studies with animals have demonstrated a down-regulation of MMPs and ADAMTS in chondrocytes by plant extracts or single constituents thereof [5, 31–33], high doses are frequently used in such settings and it is unlikely that comparable concentrations could be achieved in human patients. Importantly, when using complex plant extracts it must be kept in mind that not all components are bioavailable [34]. The present results demonstrate for the first time that bioactive components of the dietary supplement Pycnogenol® actually influence the gene expression in human articular chondrocytes in vivo.

The determination of inflammatory or metabolic factors and matrix degradation products in patients’ synovial fluid samples revealed no statistically significant differences between the study groups, but showed distinct tendencies. The mean protein concentrations of both ADAMTS-4 and ADAMTS-5 were higher in the Pycnogenol® compared to the control group. The latter is consistent with the slightly elevated ADAMTS5 gene transcription in the patients’ chondrocytes. However, the increased aggrecanase concentrations in the synovial fluid were obviously not associated with higher proteolytic activity since the main degradation product of the ADAMTSs, the sulfated glucosamine glycans (sGAG; [17]), were even slightly lower in the Pycnogenol® group. This might be explained by the fact that total ADAMTS concentrations were measured and not the aggrecanase activity. It is possible that higher ADAMTS-4 and ADAMTS-5 protein concentrations were paralleled by an increased production of the endogenous inhibitor TIMP-3 which inhibits both ADAMTSs at subnanomolar concentrations [16]. Another possibility is that post-translational processing of the ADAMTSs was not yet completed. The ADAMTSs are synthesized as zymogens and although they are assumed to be secreted as active enzymes ADAMTS-4 is further processed extracellularly to yield increased proteolytic activity [16].

In the synovial fluid MMP protein concentrations were not clearly lower in the Pycnogenol® compared to the control group. Again, this appears to be inconsistent with the observed downregulation of MMP1, MMP3 and MMP13 gene expression in the patients’ chondrocytes. The same reasoning as discussed with the ADAMTS may apply to the MMPs as well. Notably, mean concentrations of unspecific degradation products, type II collagen fragments CTX-II and Helix-II, were reduced in the Pycnogenol® group by approximately 45% and 27%, respectively, compared to controls. CTX-II is mainly produced by MMP-1, MMP-3 and MMP-13 while Helix-II is generated at high amounts by cathepsin K [21]. This suggests that despite no obvious differences in the total MMP protein concentrations an attenuated generation of collagen fragments was related to the intake of Pycnogenol®. The reduced Helix-II concentrations in the Pycnogenol® group were consistent with the slightly reduced cathepsin K gene expression in the patients’ chondrocytes.

Determination of cartilage homeostasis markers in the serum samples revealed two remarkable results. There was a significant (*p* = 0.02 and *p* = 0.001 after exclusion of a confirmed outlier) decrease of ADAMTS-5 protein concentrations in the Pycnogenol® group compared to control patients. This would be consistent with the anti-inflammatory properties and clinical effects of the pine bark extract in OA patients [7, 8, 35]. The rise of ADAMTS-5 concentrations in the control group might be explained by a further progression of joint disease or by the fact that the patients were required to refrain from anti-inflammatory analgesics. Serum levels of ADAMTS-5 have been shown to be higher in OA patients compared to healthy controls and correlate with the stage of OA [36]. The present study now provides the first evidence that serum ADAMTS-5 concentrations of OA patients decrease in response to a dietary supplement. The other finding of note is the decrease of MMP-13 protein concentrations in the Pycnogenol® group compared to control patients. The effect was not statistically significant as a consequence of the low sample numbers. Interestingly, in the Pycnogenol® group only four patients had measurable MMP-13 concentrations as opposed to seven patients in the control group. Blood levels of MMP-13 appear to be generally low. In a biomarker study with OA patients no MMP-13 was detected in a plasma preparation and only some patients had measurable levels in synovial fluid [37].

The data obtained in the present study were subjected to a correlation analysis to determine potential interrelations of biomarkers and the previously analyzed polyphenol concentrations in the respective specimen [23]. For the first time it was shown in a human study that higher gene expression levels of MMP3 in the patients’ cartilage significantly (*p* = 0.01) correlated with higher CTX-II protein levels in synovial fluid. This is consistent with in vitro results showing that MMP-3 efficiently released CTX-II [21].

A significant inverse correlation (*r* = −0.761; *p* = 0.007) was determined between MMP-3 protein levels in the patients’ synovial fluid and concentrations of ferulic acid in the same specimen. A causal relationship is likely as ferulic acid downregulates MMP3 gene expression in cells [38]. In contrast, a direct correlation between MMP-13 protein concentrations and the levels of caffeic acid in serum (*r* = 0.789; *p* = 0.02) is less explicable or supported by similar observations. A causal relationship might be assumed for the weak correlations of M1 and ADAMTS-5 concentrations in serum (*r* = −0.395, *p* = 0.016) and between M1 concentrations in blood cells and ADAMTS-4 levels in serum (*r* = −0.445, *p* = 0.033) since M1 has been previously shown to inhibit metalloproteinases [11].

The present study has some limitations. The patients in the control group did not receive a placebo. However, the current study did not aim at measuring clinical effects, e.g. symptoms reported by the patients. The aim was to investigate pharmacodynamic aspects on a cellular level as well as pharmacokinetic aspects (see [23]). To the best of our knowledge it is not possible to deliberately influence the concentration or expression of inflammatory or cartilage degradation markers. Therefore, we think that it was scientifically justified to have an untreated control group in the present pilot study. Unfortunately the variability of the obtained data was high. More pronounced group differences might have been detected with a higher number of patients. However, there was no pre-study data on the effect size of the pine bark extract on inflammatory or chondrometabolic markers in OA patients to allow for a rational calculation of the sample size. Yet, despite of the limited group sizes and the participants ignoring dietary restrictions regarding polyphenols various significant effects were uncovered. Thus, even under ‘real life’ conditions with occasional or regular consumption of dietary polyphenols the intake of Pycnogenol® had additional cellular effects in patients with severe OA. Clearly, the benefit for the patients is not the change of inflammatory or cartilage degradation markers, but the reduction of OA clinical symptoms. The latter had already been shown in clinical trials [6–8] and our study now provides a rational basis for the reported clinical effects. A strength of the current study was that more than one reference gene was used for the gene expression analysis in accordance with the MIQE guidelines [27]. Most investigations concerning cartilage gene expressions use a single housekeeping gene, particularly GAPDH, which showed high variance in expression in our hands consistent with observations of others [39] and emphasizes the importance of choosing more than one reliable reference gene. A further strength of the present investigation is that a number of biomarkers were analyzed on several levels, i.e. at both gene transcription and protein level in synovial fluid and serum. Thus, findings were substantiated by consistent observations made at different checkpoints of pathophysiologic pathways playing a role in OA.

## Conclusions

To summarize, in the present study cellular pharmacodynamic properties of Pycnogenol® were investigated in patients suffering from severe OA of the knee. For the first time the effects of a nutraceutical were systematically researched in patients’ chondrocytes, synovial fluid and serum in comparison with a control group. The overall results suggest a chondroprotective potential of the maritime pine bark extract and provide a rational basis for understanding the reported clinical effects on symptom scores in OA patients.

## Abbreviations

ACTB: β-actin
ADAMTS: a disintegrin and metalloproteinase with thrombospondin motifs
B2M: beta-2-microglobulin
COX: cyclooxigenase
CTSK: cathepsin K gene
CTX-II: carboxy-terminal telopeptides of type II collagen
DMEM: Dulbecco’s modified Eagle medium
DMMB: dimethylmethylenblue
DTT: DL-dithiothreitol
FBS: fetal bovine serum
GAPDH: glycerinaldehyde 3-phosphat dehydrogenase
Helix-II: type II collagen helical peptide
HPRT-1: hypoxanthine phosphoribosyl transferase 1
IL: interleukin
IMDM: Iscove’s modified Dulbecco’s medium
iNOS: inducible NO synthase
MIQE: minimum information for publication of quantitative real-time PCR experiments
MMP: matrix metalloproteinases
NSAID: non-steroidal anti-inflammatory drug
OA: osteoarthritis
PBS: Dulbecco’s phosphate-buffered saline
qPCR: quantitative real-time polymerase chain reaction
SDHA: succinate dehydrogenase complex, subunit A
SF: synovial fluid
sGAG: sulfated glycosaminglycans
WOMAC: Western Ontario and McMaster Universities Osteoarthritis Index
